# Broad ligament defect causing internal hernia: A case report and literature review

**DOI:** 10.1097/MD.0000000000045264

**Published:** 2025-10-24

**Authors:** Jie He, Yan Wen, Banzhu Zhang, Jun Zhou, Yuan Lin

**Affiliations:** a Department of General surgery, The Affiliated Hospital of Panzhihua University, Panzhihua, China; b Department of Obstetrics, Panzhihua Central Hospital, Panzhihua, China.

**Keywords:** broad ligament defect, case report, computer tomography, laparoscopy, small bowel obstruction

## Abstract

**Rationale:**

The incidence of intra-abdominal hernia is very low, ranging from 0.2% to 0.9% in autopsy cases and 0.5% to 4.1% in cases of intestinal obstruction. Broad ligament hernia accounts for 4% to 7% of all cases of intra-abdominal hernias.

**Patient concerns:**

A 39-year-old transient woman presented with persistent lower abdominal pain for 5 hours without any obvious cause, accompanied by anal distension and no other unusual symptoms. Initially, the patient did not undergo any intervention.

**Diagnoses:**

The computed tomography showed that the left adnexal area had a cystic low-density shadow, gas density shadow, and a close intestinal relationship, suggesting that the thickening and tortuous intestinal tube may be an adnexal source of cystic foci to be drained. Laparoscopy revealed an internal hernia caused by a broad ligament defect on the left side.

**Interventions:**

The patient was initially treated non-operatively, symptoms worsened and he was given a laparoscopic exploration with intraoperative repositioning of the bowel and repair of the defect.

**Outcomes:**

The patient started to eat on the first day after the operation when the gastrointestinal function was restored and was discharged from the hospital 3 days after the operation. In the follow-up examinations in January and June after the operation, the patient had no abdominal pain, abdominal distension, or discomfort and had normal bowel movement.

**Lessons:**

Broad ligament hernias are rare with no specific manifestations. Abdominal computer tomography is helpful for the diagnosis of a broad ligament hernia, and it should be completed in time for the timely detection of intestinal obstruction that cannot be clearly identified. Early diagnosis of hernia can be accomplished through laparoscopy to reset the hernia and repair the defect.

## 1. Introduction

Small bowel obstruction (SBO) is an obstruction of the normal flow of small bowel contents. Acute mechanical bowel obstruction is the most common type of mechanical bowel obstruction. In the United States and Western European countries, the most common cause of mechanical small bowel obstruction is intraperitoneal adhesions, followed by tumors and hernia incarceration, which accounts for approximately 90% of small bowel obstructions. The less common causes of obstruction include Crohn’s disease (1–2%), gallstones (2%), intestinal torsion (4–15%), and intussusception (4–8%).^[1]^ The incidence of intra-abdominal hernia is very low, ranging from 0.2% to 0.9% in autopsy cases and 0.5% to 4.1% in cases of intestinal obstruction. Broad ligament hernia accounts for 4% to 7% of all cases of intra-abdominal hernias.^[2]^ The broad ligament of the uterus is a peritoneal fold that connects the fallopian tubes, ovaries, and uterus to the pelvic wall and the floor. Intra-abdominal hernias through broad ligament defects are very rare and account for 4% of intra-abdominal hernias.^[3]^ This type of hernia often eponymously termed a “Quain hernia” after his discovery of SBO secondary to a broad ligament defect during an autopsy in 1861. Its pathogenesis is due to a broad ligament defect and its etiology can be congenital or acquired. The congenital cause is secondary to the spontaneous rupture of a congenital cystic structure, which is associated with abnormal pelvic peritoneal development. Acquired defects are secondary to surgical trauma, pregnancy, birth injury, pelvic inflammatory disease, or endometriosis leading to disease progression.^[4]^

## 2. Case description

This article presents a case of a 39-year-old gravida who presented to our emergency surgery department with acute abdominal pain and was recommended to be hospitalized. After treatment, her symptoms were not relieved, and she underwent emergency surgical intervention, during which she was diagnosed an internal hernia due to a broad ligament defect. In this article, we will discuss the patient’s presentation and previous examinations, and then review the latest literature on internal hernia due to broad ligament defect in the context of this case, to discuss internal hernia due to broad ligament defect, the clinical presentation of this rare disease, how to diagnose it at an early stage, how to treat it early laparoscopically, how to minimize the patient’s trauma, and how to enable rapid recovery of the patient.

**Figure 1.**
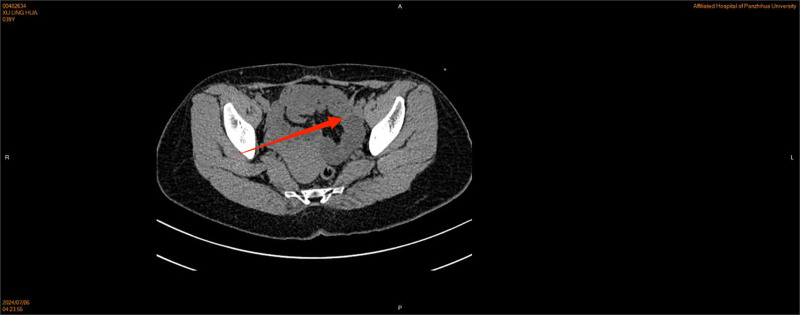
The red arrow indicates the point of obstruction.

**Figure 2.**
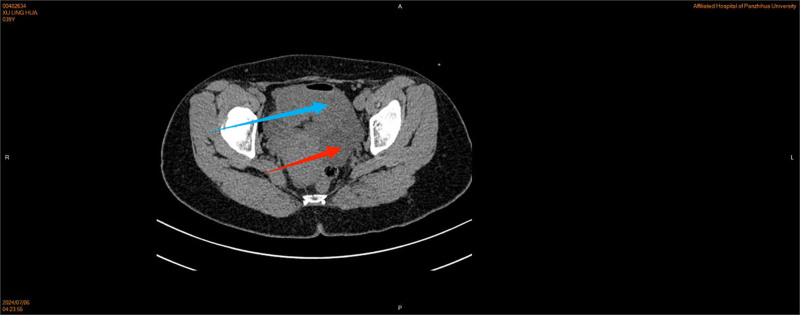
The blue arrow indicates the expansion of the intestinal tract, and the density of the 2 arrows is different.

**Figure 3.**
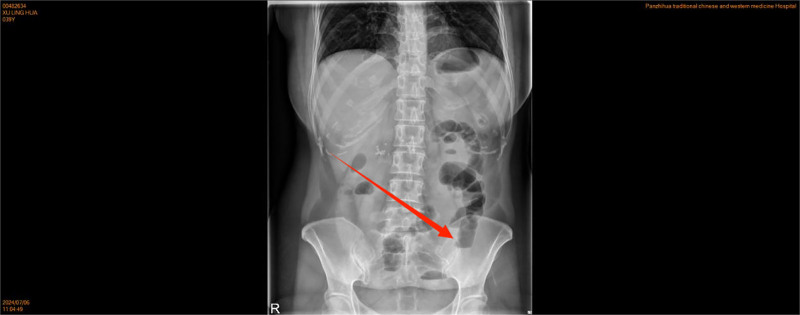
The red arrow indicates the gas-liquid level.

The patient presented with persistent lower abdominal pain for 5 hours without any obvious cause, accompanied by anal distension and no other unusual symptoms. Initially, the patient did not undergo any intervention. Four hours after the lower abdominal pain, the patient’s lower abdominal pain worsened, and the abdominal pain was persistent stabbing pain, paroxysmal aggravation, accompanied by a feeling of anal distension, lumbosacral pain, vomiting 4 to 5 times, vomiting stomach contents, small amount; during the pain period of bowel movements 2 to 3 times, small amount; no chills, fever, no vaginal bleeding, and other discomfort. The patient had a previous cesarean section in 2008, abdominal wall endometriosis lesion excision in 2010, hysteroscopic endometrial polypectomy + IUD removal in 2023, and the rest of her past and personal histories were not abnormal. Married, childbearing, G4P2, one female by normal delivery and 1 female by cesarean section. Miscarriage 2 times; the patient presented with an acute painful face, flat abdomen, soft abdomen, fixed pressure pain in the left lower abdomen, no rebound pain, muscle tension, and weak bowel sounds. Gynecological examination: Cervix: positive lifting pain, positive rocking pain, uterine pressure, soft left adnexal area with pressure pain, and normal right adnexal area. Emergency ultrasound examination showed that the right kidney stones, left kidney urinary salt crystals, and bilateral adnexal area were only partially displayed. Blood tests showed the following: white blood cell count 5.93*10^9/L, hemoglobin level 121 g/L, neutrophil percentage, 80.8%; and ultrasensitive C-reactive protein 3.6 mg/L. The urine pregnancy test results were negative. Urine test results were negative. Coagulation, hepatic and renal function, electrolytes, and preoperative combinations did not show any significant abnormalities. A preliminary diagnosis of female pelvic inflammatory disease was considered, and the obstetrics and gynecology department was admitted to carry out anti-infective treatment with a cephalosporin ornidazole combination and antispasmodic treatment with resorcinol. After 6 hours of treatment, the symptoms were not significantly relieved, and the vaginal ultrasound was reviewed again: the pelvis was about 1.4 cm deep liquid dark area; the left lower pelvis was tortuous and dilated intestinal tube-like echoes, which was “C-type,” with a wider inner diameter of about 3.2 cm, and locally dilated intestinal tubes and obstruction? Routine blood tests were normal, and computed tomography(CT) (Figs 1 and 2)showed that the left adnexal area had a cystic low-density shadow, gas density shadow, and a close intestinal relationship, suggesting that the thickening and tortuous intestinal tube may be an adnexal source of cystic foci to be drained. Abdominal standing position (Fig. 3): Fluid-gas flatness was observed in the left lower abdomen. Considering the possibility of intestinal obstruction, the patient was transferred to the general surgery treatment, scopolamine antispasmodic, cephalosporin, and ornidazole to prevent infection, enema, gastrointestinal decompression, catheterization treatment for 5 hours, a small number of bowel movements, and gastrointestinal decompression of a small amount of fluid; the symptoms were not relieved. Abdominal examination again revealed the patient’s acute painful face, flat abdomen, abdominal softness, left lower abdomen fixed pressure pain aggravation, no rebound pain muscle tension, and weak intestinal sounds. Do not rule out the possibility of narrower intestinal obstruction; communication with the patient and his family suggested laparoscopy after obtaining consent.

**Figure 4.**
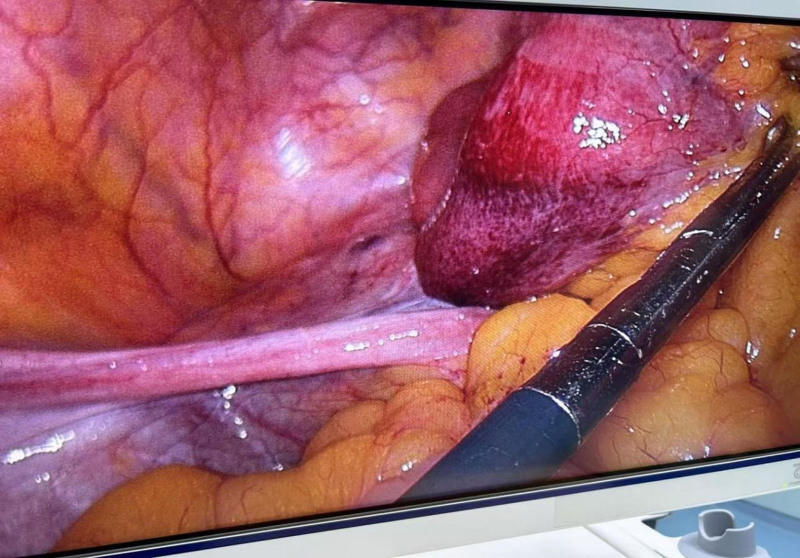
Incarcerated intestinal tube.

**Figure 5.**
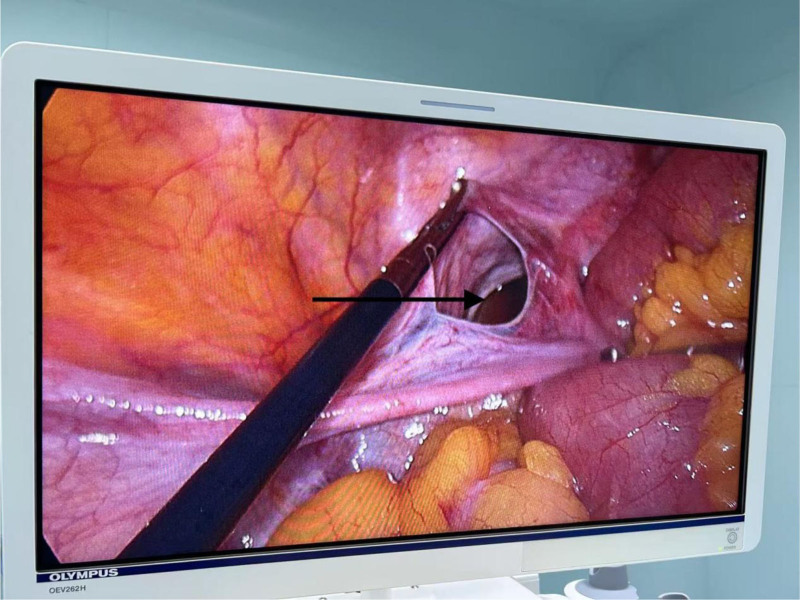
The black arrow indicates the defect area.

Intraoperatively, dilatation of the bowel was seen in the lower abdomen, approximately 50 mL of yellow ooze was seen in the pelvis, and part of the greater omentum was adhered to the pelvis. Probing from the ileocecal region, the appendix was normal, the end of the ileum was empty, along the end of the ileum, the intestinal canal was probed in the reverse direction, and approximately 10 cm of the small intestine was embedded in the left broad ligament of the uterus (Fig. 4) with a slightly dilated proximal intestinal canal filled with fluid. The herniated bowel was completely loosened, and a 5 × 4 cm laceration of the left broad ligament of the uterus was observed (Fig. 5). During the operation, the herniated bowel was not necrotic, the left broad ligament defect was closed with 3-0 absorbable suture, and the operation was completed in 30 minutes. The abdominal drainage tube was not left in place during the operation, and the poke cards were only two 5-mm and one 10-mm observation holes.

The patient started to eat on the first day after the operation when the gastrointestinal function was restored and was discharged from the hospital 3 days after the operation. In the follow-up examinations in January and June after the operation, the patient had no abdominal pain, abdominal distension, or discomfort and had normal bowel movements.

## 3. Discussion

### 3.1. Categorization

There are 2 classifications of broad ligament hernias.Hunt categorized broad ligament defects according to the extent of broad ligament defects: saccular, a defect in one layer of the broad ligament; and window, a defect in both layers of the peritoneum.Cilley categorized broad ligament hernias according to the location of the defect: type 1 defects occur caudally in the round ligament; type 2 defects occur over the round ligament of the uterus; and type 3 defects occur in the tethered membrane of the round ligament of the uterus.^[5]^ The present case was reported to be window type 2.

### 3.2. Clinical manifestations

The clinical symptoms of a broad ligament hernia vary because of the different types of broad ligament defects and symptoms caused by the different contents of the hernia. A total of 33 papers with valid data from the last 20 years were counted in Table 1,^[2–34]^ of which 2 cases in one were reported.^[21,24]^ There were 31 cases in which the hernia content was the small bowel, but hernia contents of the sigmoid colon,^[31]^ fallopian tube,^[30]^ combined fallopian tube and small bowel,^[24]^ and bladder^[33]^ were also reported. Therefore, the clinical symptoms caused by different hernia contents, varying amounts of hernia contents, and strangulation and non-strangulation differ depending on the hernia contents. When the hernia content is too high and the hernia ring is small, the clinical symptoms are severe and vice versa, and the clinical symptoms are mild. Broad ligament defects can easily occur on the left or right side, and there is no obvious pattern, according to all the statistical data that can be found in 18 cases of left broad ligament defect, 14 cases of right broad ligament defect, and 3 cases are not described; for the left side of the broad ligament defect, the incidence rate of the disease on the left side was slightly higher than that on the right side. The symptoms of the broad ligament are not specific; usually, the most common symptom is abdominal pain. The broad ligament is located in the pelvis, and according to this retrieval of clearly documented articles, there are 30 cases of patients described as lower abdominal pain, the pain is persistent and distended; one patient described as pain around the umbilicus, one patient described as abdominal distension, one patient described as epigastric pain, one patient was incidentally found intraoperatively, and one patient was found to be the result of recurrent urinary tract infections. Infection was found to be due to the bladder; 17 patients had accompanying symptoms of nausea and vomiting, and 6 patients had accompanying symptoms of anal cessation of gas and defecation. Therefore, from the collected cases, the main symptoms of broad ligament hernia are lower abdominal pain with nausea and vomiting, which are consistent with the symptoms of intestinal obstruction, such as abdominal pain, vomiting, and constipation. However, broad ligament hernia is usually a closed-loop intestinal obstruction, which is different from the common adhesive intestinal obstruction, the symptoms of which are more severe than those of adhesive intestinal obstruction, and the pain is not relieved, which may develop into intestinal perforation and intestinal necrosis if not treated in time.^[10]^

**Table 1:** Summary of studies reporting on herniation through the broad ligament over the last 20 yr.

Study	Age	Surgical history	Pregnancy history	Operation way	feature	Herniated organ(s	Diagnostic Imaging	Physic exam	Defects location
Zemour^[2]^	35	LS	N	LPR	LAP	SI	ACT	TLA	R
Anagha^[3]^	88	OT	Y	LPR	LAP, V, C	SI	ACT	TLA	R
Sajan^[4]^	51	N	Y	LPR	LAP, V, C	SI	ACT	TLA	L
Hashimoto^[5]^	53	LS	Y	LPR	LAP	SI	ACT	TLA	NM
Arif^[6]^	62	N	Y	LIRA	UAP, CV	SI	ACT	TLA	NM
Ahuja^[7]^	40	N	NM	LPR	CV	SI	ACT	TLA	L
Ishihara^[8]^	40	AP	Y	LIRA	LAP, V	SI	ACT	TLA	L
Haku^[9]^	52	AP	Y	LR	LAP, V	SI	ACT	TLA	L
Reyes^[10]^	70	LSG, RHP	Y	LR	LAP	SI	ACT	TLA	L
Nozoe^[11]^	59	AP	Y	LR	LAP, V, N	SI	ACT	NM	L
Al-Sulaimani^[12]^	42	N	Y	LIRA	LAP, V, C	SI	AX, US	RTLA	R
Mailleux^[13]^	38	N	Y	LPR	LAP	SI	ACT	TLA	R
Otani-Takei^[14]^	65	N	NM	LR	LAP, V, C	SI	AX, ACT	TLA	L
Tanioka^[15]^	49	N	Y	LR	LAP, N	SI	EN	TLA	L
Livaudais^[16]^	52	AP	Y	LIRA	LAP	SI	AX	RTLA	R
Leone^[17]^	36	N	N	LPR	LAP	SI	AX	TLA	R
Koizumi^18]^	41	N	Y	LPR	LAP	SI	ACT	TLA	R
Marzouk^[19]^	71	N	NM	LPR	LAP, V, N	SI	ACT	TLA	R
Matsunami^[20]^	36	N	N	LPR	LAP	SI	ACT	TLA	L
Agresta^[21]^	38	AP	Y	LPR	LAP, V	SI	AX\US	TLA	R
	55	N	Y	LPR	LAP, N	SI	AX\US	TLA	L
Rodrigues^[22]^	47	N	NM	LPR	AC	SI	OR	TLA	R
Song^[23]^	57	N	NM	LPIRA	LAP	SI	ACT	TLA	L
Rohatgi^[24]^	42	N	N	LPR	LAP	SI	ACT	TLA	L
	35	CN	Y	LPR	LAP	SI, AD	ACT, AX	TLA	L
Garcia-Oria^[25]^	43	AS	NM	LPR	LAP, V, N	SI	AX	TLA	L
Toolabi^[26]^	37	UHR	NM	LPR	LAP, V, N	SI	ACT\AX	TLA	L
Vyrdal^[27]^	42	N	Y	LPR	LAP, V, N	SI	ACT	TLA	R
Guillem^[28]^	33	N	Y	LPR	LAP, V	SI	ACT	TLA	NM
Bangari^[29]^	42	N	Y	LPR	LAP, V	SI	ACT	TLA	R
Demir^[30]^	42	LER	Y	LPR	LAP	AD	ACT	TLA	R
Takeyama^[31]^	52	N	NM	LPR	LAP	SC	ACT	TLA	L
Fonseca-Coronado^[32]^	36	N	NM	LPR	LAP, C	SI	ACT	TLA	L
El Madi^[33]^	13M	N	N	LR	RUI	BL	AX	RUI	L
MacDonald^[34]^	34	N	NM	AS	LAP, V, N	SI	ACT	TLA	R

AC = accident, ACT = abdomen computed tomography, AD = adnexa, AP = appendectomy, AS = abdominals surgery, AX = abdominal x-rays, C = constipation, CN = cesarean, EN = enterography, L = left, LAP = lower abdominal pain, LER = laparoscopic endometrial resection, LIRA = laparotomy, intestinal resection, intestinal anastomosis, LPR = laparoscopic release, LS = laparoscopic surgery, LSG = laparoscopic sleeve gastrectomy, N = nausea, N = NO, NM = not mentioned, OP = operaiton, OT = open tubectomy, R = right, RHP = removal of heating pipes, UHR = umbilical hernia repair, US = ultrasound, RUI = recurrent urinary tract infections, SI = small intestine, SC = sigmoid colon, TLA = tenderness in the lower abdomen, V = vomiting, Y = yes.

### 3.3. How to make early diagnosis

It is extremely important for the early recognition of intestinal obstruction due to a broad ligament defect. The broad ligament is a double layer of peritoneum that wraps around the female pelvic organs during endometrial fusion,^[35]^ and the causes of broad ligament defects are classified as congenital and acquired; therefore, there are many causes of broad ligament defects, and there is no characteristic history to aid in the diagnosis. There are no typical clinical manifestations of internal hernia due to broad ligament defects, which are usually characterized by symptoms of intestinal obstruction, such as abdominal pain, vomiting, cessation of anal evacuation, defecation, or persistent lower abdominal pain and tenderness. Literature describes^[7]^ clinical manifestations ranging from mild abdominal pain, paroxysmal colic, and intermittent pain to complete intestinal obstruction. Due to the different and uncharacteristic nature of the condition, it leads to an uncharacteristic physical examination of the patient, which may show only abdominal pressure and pain on the side of the internal hernia, no muscle tension in the early stages, and bowel sounds may be normal or hyperactive. In the present case, the patient presented with persistent pain with paroxysmal colic and fixed pressure in the left lower abdomen.

Laboratory tests will still not be characteristic, and may only show a slight increase in leukocytes and neutrophils and an increase in CRP; in this case, the patient’s laboratory tests were unremarkable. If the diagnosis is still unclear, abdominal CT should be performed as soon as possible to identify the cause of abdominal pain.CT imaging can be used to diagnose a hernia of the broad ligament, either by visualizing the mesenteric vessels crossing the defect of the broad ligament or by finding a deviation in the opposite direction of the uterus and ovaries. Color ultrasound can be used to assess the location and vascular supply of the ovaries. CT scanning was used for evaluation in 70% of cases. However, preoperative CT diagnosis of a broad ligament hernia is accurate in only 36%.^[4,7,24]^ CT diagnosis is advised frequently in the literature, with a review of CT diagnoses advocating the following diagnostic criteria: a pelvic transition point; small bowel loops dilated and herniated lateral to the uterus within the pelvic cavity; displacement of the uterus forward from the pelvic cavity; and a small bowel loop with a small pelvic cavity displacement of the uterus from the opposite side of the hernia site.^[14,34,36]^ Therefore, if we encounter unexplained lower abdominal pain in a woman who has or has not given birth, and at the same time, meets the symptoms of intestinal obstruction, abdominal pain, vomiting, anal cessation of defecation, defecation, or persistent lower abdominal pain, we need to be vigilant for the presence of an internal hernia of the broad ligament, even if the patient has no history of abdominal surgery. There was no history of abdominal surgery, especially in women who gave birth.

### 3.4. Early diagnosis, laparoscopic surgery to reduce trauma

If a broad ligament hernia can be recognized, diagnosed, and treated at an early stage, trauma to the patient will undoubtedly be minimized. In this case, the patient was found to have obstruction at the same time as perfect CT, and the abdominal signs worsened, and timely surgical laparoscopy was performed, which revealed a broad ligament hernia with obstruction, and the incarcerated intestinal tube was returned, and the defective broad ligament was repaired with sutures, to avoid further necrosis of the intestinal tube, and the cases retrieved from the review of the literature had 24 cases of incarcerated hernia return of the intestinal tube after laparoscopic exploration, and only one case of resection of intestinal tube after laparoscopic examination, one case of resection of the fallopian tubes In 11 cases, open surgery was performed, of which only 7 cases were open hernia reduction and repair, and in the remaining 4 cases, enterotomy and anastomosis were performed for necrotic intestines, and in one case, the intestines were reduced and the fallopian tube was removed.The surgical management of broad ligament hernias were first described through an open approach, but laparoscopic advancements in surgery have led to minimally invasive repair. Laparoscopy can afford improved visualization of pelvic structures and is often associated with decreased use of postoperative analgesics, decreased rate of wound complications, and decreased hospital length of stay.^[29,37]^ Even with single-incision laparoscopy, the first single-incision laparoscopic repair of a broad ligament hernia causing SBO was published by Takeyama.^[31]^ In the current literature, nearly 60% of patients undergo laparoscopy, which reduces surgical trauma, accelerates postoperative recovery, and reduces the hospitalization time. Therefore, if there is a high suspicion of broad ligament hernia, early improvement of abdominal CT to further confirm the diagnosis, if the patient is diagnosed as broad ligament hernia, early laparoscopic surgery can be completed, of course, this is also subject to the local level of medical care and surgeon’s influence!

## 4. Conclusion

A broad ligament hernia is a defect in the broad ligament that leads to herniation of the small intestine. As it has no specific manifestations and is usually similar to the symptoms of common intestinal obstruction, it cannot be diagnosed and treated in a timely manner, leading to a high mortality rate. Abdominal CT has a diagnostic accuracy of 36% for broad ligament hernias and should be completed in a timely manner for any intestinal obstruction for which the cause cannot be identified, particularly in women in labor. Early diagnosis can be accomplished laparoscopically to reposition the hernia and repair the defect, which can reduce patient trauma and speed up the patient’s rapid recovery, but it depends on local medical resources and the surgeon’s surgical experience.

## Acknowledgments

We would like to express our gratitude to the staff at the Department of General surgery, The Affiliated Hospital of Panzhihua University.

## Author contributions

**Conceptualization:** Jie He, Yan Wen.

**Data curation:** Jun Zhou.

**Formal analysis:** Jie He, Banzhu Zhang, Jun Zhou, Yuan Lin.

**Funding acquisition:** Jie He.

**Investigation:** Banzhu Zhang, Jun Zhou.

**Methodology:** Jie He, Banzhu Zhang.

**Project administration:** Jie He.

**Resources:** Jie He.

**Software:** Yan Wen.

**Supervision:** Jie He.

**Visualization:** Yan Wen.

**Writing – original draft:** Jie He.

**Writing – review & editing:** Jie He, Yan Wen.
